# Bis(4-eth­oxy­phen­yl) sulfoxide

**DOI:** 10.1107/S1600536811013213

**Published:** 2011-04-16

**Authors:** Kang Meng, Cheng Wu, Jing Cao, Ping Ma, Lihong Liu

**Affiliations:** a Pharmacy Department of the Second Artillery General Hospital, Beijing 100088, People’s Republic of China

## Abstract

In the title compound, C_16_H_18_O_3_S, the dihedral angle between the benzene rings is 82.7 (2)°. The O atom of the sulfoxide group is disordered over two orientations with refined occupancy factors of 0.563 (3):0.437 (3). In the crystal, mol­ecules are linked by inter­molecular C—H⋯O hydrogen bonds, forming chains along the *b* axis.

## Related literature

For background to Friedel–Crafts acyl­ation, see: Edward & Sibelle (1963 ▶); DeHaan *et al.* (1979 ▶); Fillion & Fishlock (2005 ▶); Nishimoto *et al.* (2008 ▶). For the structures of related aryl­sulfoxides, see: Casarini *et al.* (2004 ▶); Noland & Kedrowski (2000 ▶).
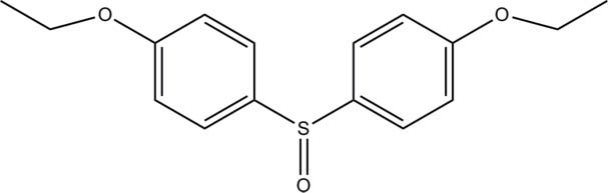


## Experimental

### 

#### Crystal data

C_16_H_18_O_3_S
*M* *_r_* = 290.36
Triclinic, 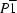

*a* = 8.2052 (16) Å
*b* = 9.856 (2) Å
*c* = 10.196 (2) Å
α = 64.71 (3)°
β = 83.78 (3)°
γ = 82.88 (3)°
*V* = 738.4 (3) Å^3^
*Z* = 2
Mo *K*α radiation
μ = 0.22 mm^−1^
*T* = 113 K
0.20 × 0.16 × 0.12 mm

#### Data collection

Rigaku Saturn CCD area-detector diffractometer
Absorption correction: multi-scan (*CrystalClear*; Rigaku, 2007 ▶) *T*_min_ = 0.957, *T*_max_ = 0.974
6624 measured reflections
3450 independent reflections
2590 reflections with *I* > 2σ(*I*)
*R*_int_ = 0.022

#### Refinement

*R*[*F* ^2^ > 2σ(*F* ^2^)] = 0.034
*wR*(*F* ^2^) = 0.095
*S* = 1.06
3450 reflections
191 parameters
H-atom parameters constrained
Δρ_max_ = 0.26 e Å^−3^
Δρ_min_ = −0.40 e Å^−3^

### 

Data collection: *CrystalClear* (Rigaku, 2007 ▶); cell refinement: *CrystalClear*; data reduction: *CrystalClear*; program(s) used to solve structure: *SHELXS97* (Sheldrick, 2008 ▶); program(s) used to refine structure: *SHELXL97* (Sheldrick, 2008 ▶); molecular graphics: *SHELXTL* (Sheldrick, 2008 ▶); software used to prepare material for publication: *SHELXTL*.

## Supplementary Material

Crystal structure: contains datablocks I, global. DOI: 10.1107/S1600536811013213/rz2577sup1.cif

Structure factors: contains datablocks I. DOI: 10.1107/S1600536811013213/rz2577Isup2.hkl

Additional supplementary materials:  crystallographic information; 3D view; checkCIF report

## Figures and Tables

**Table 1:** Hydrogen-bond geometry (Å, °)

*D*—H⋯*A*	*D*—H	H⋯*A*	*D*⋯*A*	*D*—H⋯*A*
C11—H11⋯O1^i^	0.93	2.51	3.3013 (18)	143

Symmetry code: (i) 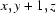.

**Fig. 1.**
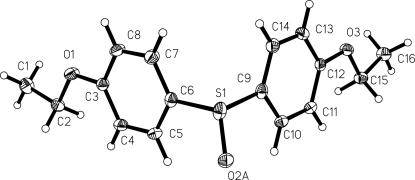
The molecular structure of the title compound, with 40% probability displacement ellipsoids. Only the major component of disorder is shown.

## supplementary crystallographic information

### Comment

Friedel-Crafts acylation is a potent method for building acylbenzene derivatives (Edward & Sibelle, 1963; DeHaan *et al.*, 1979; Fillion & Fishlock, 2005; Nishimoto *et al.*, 2008). Thiocarbonylbenzenes may be prepared by Friedel-Crafts acylation of benzene derivatives with thiocarbonyl chloride in the presence of anhydrous aluminium chloride. Thus, in order to investigate the potentiality of the method, the title compound was prepared by Friedel-Crafts acylation of phenetol, an electron-rich benzene derivative.

In the title compound (Fig. 1) the dihedral angle between the two benzene rings (C3—C8 and C9—C14) is 82.7 (2)°. The S=O bond length is shorter than those found in previously reported arylsulfoxides (Casarini *et al.*, 2004; Noland & Kedrowski, 2000). The oxygen atom of the sulfoxide group is disordered over two orientations with site occupancies of 0.563 (3) and 0.437 (3) for the major and minor components, repectively. In the crystal structure, molecules are linkied by intermolecular C—H···O hydrogen bonds (Table 1) to form chains along the *b* axis.

### Experimental

A round-bottomed flask was charged with 1.19 g (10 mmol) of freshly distilled thionyl chloride, 2.44 g (20 mmol) of phenetol and 20 ml of dried dichloromethane, and the mixture was stirred on an ice-water bath followed by addition of 2.67 g (20 mmol) of anhydrous aluminium chloride in a portionwise manner. The resulting mixture was stirred at room temperature overnight and poured into 200 ml of ice-water. The mixture thus formed was exacted with three 50-ml portions of dichloromethane, and the combined exacts were washed with saturated brine, dried over sodium sulfate and evaporated on a rotary evaporator to afford the crude title compound. Pure title compound was obtained by column chromatography. Crystals suitable for X-ray diffraction were obtained through slow evaporation of a ethyl acetate/petroleum ether (1:10 *v*/*v*) solution.

### Refinement

H atoms were positioned geometrically and refined using a riding model, with C—H = 0.93–0.97 Å, and with *U*_iso_(H) = 1.2 *U*_eq_(C) or 1.5 *U*_eq_(C) for methyl H atoms.

### Crystal data

**Table:**

C_16_H_18_O_3_S	*Z* = 2
*M**_r_* = 290.36	*F*(000) = 308
Triclinic, *P*1	*D*_x_ = 1.306 Mg m^−^^3^
Hall symbol: -P 1	Mo *K*α radiation, λ = 0.71073 Å
*a* = 8.2052 (16) Å	Cell parameters from 2289 reflections
*b* = 9.856 (2) Å	θ = 2.2–27.9°
*c* = 10.196 (2) Å	µ = 0.22 mm^−^^1^
α = 64.71 (3)°	*T* = 113 K
β = 83.78 (3)°	Block, colourless
γ = 82.88 (3)°	0.20 × 0.16 × 0.12 mm
*V* = 738.4 (3) Å^3^	

### Data collection

**Table:**

Rigaku Saturn CCD area-detector diffractometer	3450 independent reflections
Radiation source: rotating anode	2590 reflections with *I* > 2σ(*I*)
confocal	*R*_int_ = 0.022
Detector resolution: 7.31 pixels mm^-1^	θ_max_ = 27.9°, θ_min_ = 2.2°
ω and φ scans	*h* = −10→10
Absorption correction: multi-scan (*CrystalClear*; Rigaku, 2007)	*k* = −12→9
*T*_min_ = 0.957, *T*_max_ = 0.974	*l* = −13→12
6624 measured reflections	

### Refinement

**Table:**

Refinement on *F*^2^	Primary atom site location: structure-invariant direct methods
Least-squares matrix: full	Secondary atom site location: difference Fourier map
*R*[*F*^2^ > 2σ(*F*^2^)] = 0.034	Hydrogen site location: inferred from neighbouring sites
*wR*(*F*^2^) = 0.095	H-atom parameters constrained
*S* = 1.06	*w* = 1/[σ^2^(*F*_o_^2^) + (0.0422*P*)^2^ + 0.2002*P*] where *P* = (*F*_o_^2^ + 2*F*_c_^2^)/3
3450 reflections	(Δ/σ)_max_ = 0.001
191 parameters	Δρ_max_ = 0.26 e Å^−^^3^
0 restraints	Δρ_min_ = −0.40 e Å^−^^3^

### Special details

**Table:**

Geometry. All e.s.d.'s (except the e.s.d. in the dihedral angle between two l.s. planes) are estimated using the full covariance matrix. The cell e.s.d.'s are taken into account individually in the estimation of e.s.d.'s in distances, angles and torsion angles; correlations between e.s.d.'s in cell parameters are only used when they are defined by crystal symmetry. An approximate (isotropic) treatment of cell e.s.d.'s is used for estimating e.s.d.'s involving l.s. planes.
Refinement. Refinement of *F*^2^ against ALL reflections. The weighted *R*-factor *wR* and goodness of fit *S* are based on *F*^2^, conventional *R*-factors *R* are based on *F*, with *F* set to zero for negative *F*^2^. The threshold expression of *F*^2^ > σ(*F*^2^) is used only for calculating *R*-factors(gt) *etc*. and is not relevant to the choice of reflections for refinement. *R*-factors based on *F*^2^ are statistically about twice as large as those based on *F*, and *R*- factors based on ALL data will be even larger.

### Fractional atomic coordinates and isotropic or equivalent isotropic displacement parameters (Å^2^)

**Table:**

	*x*	*y*	*z*	*U*_iso_*/*U*_eq_	Occ. (<1)
S1	−0.00304 (4)	0.50094 (4)	0.73977 (4)	0.02589 (12)	
O1	0.32089 (14)	−0.11895 (11)	0.97342 (11)	0.0264 (2)	
O2A	−0.0884 (2)	0.5328 (2)	0.8531 (2)	0.0284 (5)	0.563 (3)
O2B	−0.0815 (3)	0.5217 (3)	0.6188 (3)	0.0274 (7)	0.437 (3)
O3	0.60436 (12)	0.81887 (11)	0.56357 (10)	0.0237 (2)	
C1	0.4363 (2)	−0.35101 (18)	1.14478 (18)	0.0316 (4)	
H1A	0.4553	−0.4098	1.2457	0.047*	
H1B	0.5396	−0.3364	1.0887	0.047*	
H1C	0.3694	−0.4028	1.1126	0.047*	
C2	0.34927 (19)	−0.20046 (16)	1.12487 (15)	0.0248 (3)	
H2A	0.2456	−0.2138	1.1827	0.030*	
H2B	0.4167	−0.1460	1.1551	0.030*	
C3	0.24360 (17)	0.02257 (15)	0.92796 (15)	0.0205 (3)	
C4	0.18830 (19)	0.09277 (17)	1.01871 (16)	0.0247 (3)	
H4	0.2018	0.0426	1.1180	0.030*	
C5	0.11249 (18)	0.23851 (17)	0.96065 (16)	0.0252 (3)	
H5	0.0752	0.2862	1.0211	0.030*	
C6	0.09267 (17)	0.31234 (15)	0.81312 (16)	0.0211 (3)	
C7	0.14640 (19)	0.24226 (17)	0.72137 (16)	0.0261 (3)	
H7	0.1315	0.2921	0.6223	0.031*	
C8	0.2223 (2)	0.09749 (17)	0.77930 (16)	0.0259 (3)	
H8	0.2592	0.0499	0.7188	0.031*	
C9	0.17993 (17)	0.59829 (15)	0.68666 (16)	0.0217 (3)	
C10	0.23615 (18)	0.64787 (16)	0.78032 (16)	0.0223 (3)	
H10	0.1784	0.6310	0.8688	0.027*	
C11	0.37828 (17)	0.72262 (15)	0.74289 (15)	0.0211 (3)	
H11	0.4161	0.7561	0.8057	0.025*	
C12	0.46333 (17)	0.74683 (15)	0.61065 (15)	0.0191 (3)	
C13	0.40644 (19)	0.69756 (16)	0.51575 (15)	0.0240 (3)	
H13	0.4643	0.7141	0.4274	0.029*	
C14	0.26404 (18)	0.62420 (16)	0.55302 (16)	0.0240 (3)	
H14	0.2248	0.5924	0.4895	0.029*	
C15	0.67526 (18)	0.85931 (17)	0.66294 (16)	0.0235 (3)	
H15A	0.6960	0.7712	0.7529	0.028*	
H15B	0.6014	0.9326	0.6850	0.028*	
C16	0.83409 (18)	0.92527 (18)	0.58926 (17)	0.0278 (3)	
H16A	0.8900	0.9472	0.6550	0.042*	
H16B	0.8110	1.0164	0.5043	0.042*	
H16C	0.9025	0.8543	0.5616	0.042*	

### Atomic displacement parameters (Å^2^)

**Table:**

	*U*^11^	*U*^22^	*U*^33^	*U*^12^	*U*^13^	*U*^23^
S1	0.01736 (17)	0.02001 (18)	0.0400 (2)	0.00022 (12)	−0.00217 (15)	−0.01266 (16)
O1	0.0373 (6)	0.0221 (5)	0.0180 (5)	0.0067 (4)	−0.0050 (4)	−0.0082 (4)
O2A	0.0233 (10)	0.0257 (10)	0.0363 (12)	−0.0013 (7)	0.0091 (8)	−0.0159 (9)
O2B	0.0276 (13)	0.0241 (13)	0.0301 (14)	−0.0007 (10)	−0.0155 (10)	−0.0081 (11)
O3	0.0258 (5)	0.0279 (6)	0.0200 (5)	−0.0089 (4)	0.0021 (4)	−0.0116 (4)
C1	0.0339 (9)	0.0257 (8)	0.0287 (9)	0.0021 (6)	−0.0069 (7)	−0.0054 (7)
C2	0.0304 (8)	0.0239 (7)	0.0173 (7)	−0.0016 (6)	−0.0049 (6)	−0.0054 (6)
C3	0.0223 (7)	0.0193 (7)	0.0199 (7)	−0.0006 (5)	−0.0017 (5)	−0.0082 (6)
C4	0.0310 (8)	0.0258 (8)	0.0171 (7)	−0.0021 (6)	0.0006 (6)	−0.0093 (6)
C5	0.0278 (8)	0.0259 (8)	0.0245 (8)	−0.0015 (6)	0.0049 (6)	−0.0150 (6)
C6	0.0161 (6)	0.0194 (7)	0.0279 (8)	−0.0025 (5)	−0.0005 (5)	−0.0101 (6)
C7	0.0338 (8)	0.0233 (7)	0.0210 (7)	0.0008 (6)	−0.0064 (6)	−0.0089 (6)
C8	0.0360 (8)	0.0239 (7)	0.0201 (7)	0.0036 (6)	−0.0034 (6)	−0.0127 (6)
C9	0.0189 (7)	0.0163 (6)	0.0284 (8)	0.0012 (5)	−0.0031 (6)	−0.0081 (6)
C10	0.0217 (7)	0.0224 (7)	0.0240 (7)	0.0001 (5)	0.0027 (6)	−0.0124 (6)
C11	0.0229 (7)	0.0216 (7)	0.0218 (7)	−0.0009 (5)	−0.0012 (6)	−0.0124 (6)
C12	0.0212 (7)	0.0154 (6)	0.0195 (7)	−0.0012 (5)	−0.0017 (5)	−0.0061 (5)
C13	0.0302 (8)	0.0241 (7)	0.0171 (7)	−0.0043 (6)	−0.0010 (6)	−0.0076 (6)
C14	0.0301 (8)	0.0219 (7)	0.0213 (7)	−0.0029 (6)	−0.0066 (6)	−0.0090 (6)
C15	0.0221 (7)	0.0291 (8)	0.0225 (7)	−0.0045 (6)	−0.0014 (6)	−0.0134 (6)
C16	0.0231 (7)	0.0342 (8)	0.0285 (8)	−0.0065 (6)	−0.0001 (6)	−0.0149 (7)

### Geometric parameters (Å, °)

**Table:**

S1—O2B	1.379 (2)	C6—C7	1.391 (2)
S1—O2A	1.4156 (18)	C7—C8	1.383 (2)
S1—C9	1.7902 (15)	C7—H7	0.9300
S1—C6	1.7913 (16)	C8—H8	0.9300
O1—C3	1.3619 (17)	C9—C10	1.383 (2)
O1—C2	1.4338 (17)	C9—C14	1.393 (2)
O3—C12	1.3659 (17)	C10—C11	1.3873 (19)
O3—C15	1.4331 (16)	C10—H10	0.9300
C1—C2	1.505 (2)	C11—C12	1.387 (2)
C1—H1A	0.9600	C11—H11	0.9300
C1—H1B	0.9600	C12—C13	1.3949 (19)
C1—H1C	0.9600	C13—C14	1.382 (2)
C2—H2A	0.9700	C13—H13	0.9300
C2—H2B	0.9700	C14—H14	0.9300
C3—C4	1.385 (2)	C15—C16	1.505 (2)
C3—C8	1.394 (2)	C15—H15A	0.9700
C4—C5	1.390 (2)	C15—H15B	0.9700
C4—H4	0.9300	C16—H16A	0.9600
C5—C6	1.381 (2)	C16—H16B	0.9600
C5—H5	0.9300	C16—H16C	0.9600
			
O2B—S1—O2A	120.99 (14)	C6—C7—H7	120.4
O2B—S1—C9	110.29 (12)	C7—C8—C3	120.34 (13)
O2A—S1—C9	107.71 (9)	C7—C8—H8	119.8
O2B—S1—C6	107.72 (11)	C3—C8—H8	119.8
O2A—S1—C6	109.65 (10)	C10—C9—C14	120.52 (13)
C9—S1—C6	98.07 (7)	C10—C9—S1	118.90 (11)
C3—O1—C2	118.08 (11)	C14—C9—S1	120.58 (11)
C12—O3—C15	117.39 (11)	C9—C10—C11	120.32 (13)
C2—C1—H1A	109.5	C9—C10—H10	119.8
C2—C1—H1B	109.5	C11—C10—H10	119.8
H1A—C1—H1B	109.5	C12—C11—C10	119.24 (13)
C2—C1—H1C	109.5	C12—C11—H11	120.4
H1A—C1—H1C	109.5	C10—C11—H11	120.4
H1B—C1—H1C	109.5	O3—C12—C11	123.89 (12)
O1—C2—C1	107.09 (12)	O3—C12—C13	115.57 (12)
O1—C2—H2A	110.3	C11—C12—C13	120.54 (13)
C1—C2—H2A	110.3	C14—C13—C12	120.01 (13)
O1—C2—H2B	110.3	C14—C13—H13	120.0
C1—C2—H2B	110.3	C12—C13—H13	120.0
H2A—C2—H2B	108.6	C13—C14—C9	119.37 (13)
O1—C3—C4	124.36 (13)	C13—C14—H14	120.3
O1—C3—C8	115.56 (12)	C9—C14—H14	120.3
C4—C3—C8	120.08 (13)	O3—C15—C16	106.61 (11)
C3—C4—C5	119.69 (13)	O3—C15—H15A	110.4
C3—C4—H4	120.2	C16—C15—H15A	110.4
C5—C4—H4	120.2	O3—C15—H15B	110.4
C6—C5—C4	119.88 (13)	C16—C15—H15B	110.4
C6—C5—H5	120.1	H15A—C15—H15B	108.6
C4—C5—H5	120.1	C15—C16—H16A	109.5
C5—C6—C7	120.83 (14)	C15—C16—H16B	109.5
C5—C6—S1	119.31 (11)	H16A—C16—H16B	109.5
C7—C6—S1	119.86 (12)	C15—C16—H16C	109.5
C8—C7—C6	119.17 (14)	H16A—C16—H16C	109.5
C8—C7—H7	120.4	H16B—C16—H16C	109.5
			
C3—O1—C2—C1	−179.66 (12)	O2B—S1—C9—C10	152.14 (15)
C2—O1—C3—C4	−0.9 (2)	O2A—S1—C9—C10	18.17 (15)
C2—O1—C3—C8	178.78 (13)	C6—S1—C9—C10	−95.51 (12)
O1—C3—C4—C5	179.25 (13)	O2B—S1—C9—C14	−27.63 (17)
C8—C3—C4—C5	−0.4 (2)	O2A—S1—C9—C14	−161.60 (13)
C3—C4—C5—C6	0.0 (2)	C6—S1—C9—C14	84.72 (13)
C4—C5—C6—C7	0.6 (2)	C14—C9—C10—C11	−0.7 (2)
C4—C5—C6—S1	−179.25 (11)	S1—C9—C10—C11	179.49 (11)
O2B—S1—C6—C5	−150.37 (15)	C9—C10—C11—C12	−0.1 (2)
O2A—S1—C6—C5	−16.90 (15)	C15—O3—C12—C11	5.76 (19)
C9—S1—C6—C5	95.24 (13)	C15—O3—C12—C13	−174.52 (12)
O2B—S1—C6—C7	29.81 (17)	C10—C11—C12—O3	−179.91 (13)
O2A—S1—C6—C7	163.29 (13)	C10—C11—C12—C13	0.4 (2)
C9—S1—C6—C7	−84.58 (13)	O3—C12—C13—C14	−179.60 (13)
C5—C6—C7—C8	−0.8 (2)	C11—C12—C13—C14	0.1 (2)
S1—C6—C7—C8	179.03 (11)	C12—C13—C14—C9	−0.9 (2)
C6—C7—C8—C3	0.4 (2)	C10—C9—C14—C13	1.2 (2)
O1—C3—C8—C7	−179.49 (13)	S1—C9—C14—C13	−178.98 (11)
C4—C3—C8—C7	0.2 (2)	C12—O3—C15—C16	175.76 (12)

### Hydrogen-bond geometry (Å, °)

**Table:**

*D*—H···*A*	*D*—H	H···*A*	*D*···*A*	*D*—H···*A*
C11—H11···O1^i^	0.93	2.51	3.3013 (18)	143

Symmetry codes: (i) *x*, *y*+1, *z*.
